# Special Issue “Natural Product-Based Strategy for Skin Inflammatory Disorders and Regeneration”

**DOI:** 10.3390/ijms27136017

**Published:** 2026-07-04

**Authors:** Ji-Hye Kang, Mi-Young Lee

**Affiliations:** 1 Department of Medical Biotechnology, College of Medical Science, Soonchunhyang University, Asan 31538, Chungnam, Republic of Korea; addio9187@sch.ac.kr; 2 Department of Medical Science, College of Medical Science, Soonchunhyang University, Asan 31538, Chungnam, Republic of Korea

Natural products have a long history of use as valuable therapeutic resources, and continue to provide structurally diverse bioactive compounds for pharmaceutical, biomedical, nutraceutical, and cosmetic applications. These materials include plant-, animal-, marine-, microbial-, and mineral-derived compounds, as well as naturally occurring biomolecules produced by living systems [1,2]. Their chemical diversity, ranging from polyphenols, flavonoids, alkaloids, terpenoids, and glycosides to complex biological extracts, provides a broad basis for modulating skin physiology and pathology [3,4].

The skin is a highly dynamic barrier organ that must continuously respond to ultraviolet radiation, microbial exposure, oxidative stress, allergens, and mechanical injury. In chronic inflammatory skin disorders and impaired wound repair, excessive inflammatory signaling, immune dysregulation, reactive oxygen species (ROS) accumulation, extracellular matrix degradation, and delayed re-epithelialization frequently interact to perpetuate tissue damage [5,6]. Natural product-based strategies are therefore particularly attractive because they often act through multiple complementary mechanisms, including antioxidant defense, inflammatory pathway modulation, collagen and elastin regulation, barrier support, angiogenesis, and stimulation of cellular proliferation and migration [5,7,8,9]. Nevertheless, rigorous standardization, mechanistic validation, safety assessment, and clinical translation remain essential to convert promising natural materials into reliable dermatological interventions [5,9].

This Special Issue brings together six original research articles and one review article under the topic of “Natural Product-Based Strategy for Skin Inflammatory Disorders and Regeneration”. Collectively, these contributions address natural product-derived approaches for controlling inflammation, protecting against UV-induced damage, improving skin hydration and aging-related changes, and promoting wound healing and regeneration.

Several contributions focus on inflammatory regulation and the functional enhancement of plant-derived materials. *Paspalum thunbergii*, a perennial herb native to Korea, exhibited potent anti-inflammatory activity in LPS-stimulated RAW 264.7 macrophages (Contribution 1). The extract contained multiple LC-MS-identified constituents, including rosmarinic acid and isoquercitrin, showed radical-scavenging activity, and suppressed LPS-induced nitric oxide production as well as iNOS and COX-2 expression. Mechanistically, its activity was linked to the coordinated inhibition of MAPK, NF-κB, JAK/STAT, and Wnt/β-catenin signaling pathways. In another study, the fermentation of Snowberry (*Symphoricarpos albus*) extracts with *Lactobacillus plantarum* altered the phenolic profile of the plant material, particularly by increasing ferulic acid in fermented leaf and fruit extracts (Contribution 2). These biochemical changes were associated with increased expression of skin hydration-related genes AQP3 and HAS3 and the collagen synthesis gene COL1A1, supporting the potential of fermented botanical extracts as natural ingredients for skin hydration, anti-wrinkle activity, and healthy aging.

Photoprotection emerges as another major theme of this Special Issue. Thua Nao, a traditional Thai fermented soybean product, protected UVB-exposed HaCaT keratinocytes from oxidative damage, inflammation, and apoptosis (Contribution 3). Its dichloromethane fraction and the isoflavones daidzein and glycitein reduced intracellular ROS, enhanced antioxidant enzymes such as superoxide dismutase and glutathione peroxidase 4, inhibited caspase-9 and caspase-3 activation, and modulated MAPK/Akt signaling. Complementing these cell-based findings, a botanical blend of rosemary and grapefruit extracts (Nutroxsun^®^) was evaluated using both in vitro models and a placebo-controlled, randomized, crossover human study (Contribution 4). The formulation reduced ROS generation and proinflammatory interleukins in UV-irradiated keratinocytes, preserved extracellular matrix-related components in fibroblasts by supporting procollagen I and elastin while reducing MMP-1 and MMP-3, and significantly attenuated UV-induced erythema in human subjects. These findings highlight the translational promise of oral natural product-based interventions for photoprotection and photoaging management.

Beyond inflammation and photoprotection, natural products were also investigated as drivers of skin repair. *Haberlea rhodopensis*, a rare “resurrection plant” endemic to Greece and Bulgaria and known for its extreme desiccation tolerance and rich phytochemical composition, was evaluated for antioxidant, cytoprotective, and wound-healing-associated activity in HaCaT keratinocytes (Contribution 5). Its ethanolic extract protected plasmid DNA from Fenton-reaction-induced oxidative damage, maintained keratinocyte viability under H_2_O_2_-induced stress, reduced intracellular ROS generation, modulated antioxidant-related genes such as CAT, SOD1, HMOX1, and NQO1, GPX, GSR, and enhanced scratch wound closure. These results support the relevance of stress-tolerant medicinal plants as sources of skin-protective and repair-promoting agents.

The review article in this Special Issue further consolidates the mechanistic rationale for polyphenol-based dermatological interventions (Contribution 6). Proanthocyanidins (PACs), a subclass of polyphenolic compounds, are highlighted as promising agents for inflammation-related skin disorders and impaired wound healing. The review summarizes evidence that PACs can promote wound closure through pro-regenerative, pro-angiogenic, antioxidative, anti-inflammatory, and collagen-regulating activities, while also showing potential for mitigating atopic dermatitis, psoriasis, and UV-induced dermatitis. Importantly, the review also emphasized remaining gaps, including the need for clinical verification, clearer structure–activity relationships, and optimized topical delivery systems.

Finally, this Special Issue broadens the concept of natural product-based dermatological regeneration by introducing a cell-free regenerative medicine strategy utilizing mesenchymal stem cell-derived extract (MSC-ext) for skin wound healing (Contribution 7). Although MSC-ext is not a conventional botanical extract, it represents an important category of naturally derived bioactive material produced by living cells. Mesenchymal stem cell-derived factors contain a coordinated repertoire of endogenous humoral mediators that can influence proliferation, migration, angiogenesis, extracellular matrix remodeling, and tissue maturation. In the highlighted study, MSC-ext enhanced the proliferation of dermal fibroblasts, epithelial cells, and endothelial cells; increased the migration of fibroblasts and epithelial cells; and accelerated wound closure in a mouse skin defect model. Histological findings, including thicker dermis, larger Picrosirius red-positive areas, and increased Ki67-positive cells, further demonstrated its strong regenerative efficacy. These findings suggest that mesenchymal-stem-cell-derived extracts can complement phytochemical- and plant-extract-based approaches by providing a potent, cell-free, and biologically integrated platform for skin regeneration. As a natural reservoir of multiple pro-regenerative cues rather than a single isolated compound, MSC-ext may be especially valuable for complex wounds in which inflammation, matrix deposition, angiogenesis, and re-epithelialization must be coordinated.

Taken together, the articles in this Special Issue demonstrate the expanding therapeutic potential of natural product-based strategies in dermatology. The collected studies span anti-inflammatory phytochemicals, fermented botanical extracts, UV-protective dietary ingredients, stress-adapted medicinal plants, polyphenolic therapeutic candidates, and cell-derived regenerative extracts. Despite their diversity, these approaches converge on shared biological mechanisms: suppression of oxidative stress and inflammatory mediators, preservation of extracellular matrix integrity, enhancement of hydration- and collagen-related gene expression, support of angiogenesis, and stimulation of cell proliferation and migration. Future studies should emphasize chemical and biological standardization, dose–response characterization, formulation optimization, long-term safety profiling, and well-designed clinical studies. Such efforts will be essential for translating natural product-based discoveries into evidence-based strategies for inflammatory skin disorders, skin aging, wound healing, and tissue regeneration.
